# Creative Processes in the Shaping of a Musical Interpretation: A Study of Nine Professional Musicians

**DOI:** 10.3389/fpsyg.2018.00665

**Published:** 2018-05-08

**Authors:** Isabelle Héroux

**Affiliations:** Groupe de Recherche Interdisciplinaire sur les Arts Vivants, Département de Musique, Université du Québec à Montréal, Montréal, QC, Canada

**Keywords:** creative process, musician, expert, music, interpretation, practice, creativity

## Abstract

Various studies have been conducted to understand the role of mental representation when musicians practice or perform music (Lehman and Ericsson, 1997; Sloboda, 2005) and the work steps required for a musician to prepare a concert (Chaffin et al., 2003). More recent studies examine creativity in the shaping of a musical interpretation (Lisboa et al., 2011; Payne, 2016; Barros et al., 2017; Wise et al., 2017). However, none of these studies answers the following questions: Why do expert musicians working from the same score create different musical interpretations? During individual practice sessions, what happens that allows each musician to produce significantly different interpretive results? To answer these questions, we instructed nine expert musicians to record their individual practice sessions, verbalize their actions and thoughts, and answer a self-reflection questionnaire. A third-party observer also described what happened during the practice sessions. We conducted interviews in order to gather additional information about the contents of the individual practice sessions; the musicians' usual work habits; and their beliefs, values, and ideas regarding the role of the musician in the creative process. Based on the methodology of *Analyse par théorisation ancrée*^1^ (Paillé, 1994), we were able to take into account a diverse data set and identify aspects of the creative process that were specific to each individual as well as elements that all musicians shared. We found that the context in which the creative process takes place—the musician (e.g., his or her values and knowledge); the musical work (e.g., style, technical aspects, etc.); and the external constraints (e.g., deadlines, public expectations, etc.)—impacted the strategies used. The participants used reflection, extramusical supports, emotions, body reactions, intuition, and other tools to generate new musical ideas and evaluate the accuracy of their musical interpretations. We identified elements related to those already discussed in the literature, including the creative process as an alternation between divergent and convergent thinking (Guilford, 1950), creative associations (Lubart, 2015), and artistic appropriation (Héroux and Fortier, 2014; Héroux, 2016).

## Introduction

In 1950, Guilford wrote that creativity “represents an area in which psychologists generally, whether they be angels or not, have feared to tread” (Guilford, 1950, p. 444). Times have changed, however. Numerous studies have been conducted, leading to a variety of theories of the creative process. These theories may be classified according to type and orientation^2^ with respect to a specific area of creative practice, or across several areas. Contrary to Stein (1953), who criticized research focusing only on genius, present-day researchers have explored the gamut of creative practice, from the “mini-c” of everyday creativity to the “Big C” of world-class artists. Their work explores various facets of the creative process, most notably the “Six P's” of creativity: Product (artistic output and inventions), Process (the mental mechanisms involved in creative thinking), Persons (individual traits of creative persons), Place (environments that allow creativity to bloom), and, more recently, Persuasion (the capacity of an individual to influence his or her domain, i.e., the social aspect of creativity) and Potential (a developmental approach to creative studies) (Kozbelt et al., 2010).

Studies of creativity typically concentrate on general aspects of creativity common to all artistic domains or on elements particular to a given domain (Kaufman and Baer, 2005; Kaufman and Sternberg, 2010). Creativity in music performance was first studied with respect to improvisation (Clarke, 1988, 2012; Pressing, 1988; Kenny and Gellrich, 2002) and, more recently, with respect to music interpretation (Lisboa et al., 2011; Héroux and Fortier, 2014; Héroux, 2016; Payne, 2016; Barros et al., 2017; Wise et al., 2017). As Clarke (2012) notes, identifying creative elements in the interpretation of written music is not an easy task. Music interpretation is ruled by strong stylistic and instrumental constraints: accepted approaches to playing a specific repertoire and a specific instrument condition a performer's musical output.

Music does not exist in a vacuum: performers interpret pieces within a cultural framework of performance traditions. These traditions offer guidelines for acceptable playing, ranging from score reading to appropriate physical techniques. They are largely unwritten, disseminated orally (and aurally) by teachers and performers. Although these standards regulate “good” vs. “bad” playing, they are not universal: performers continually adapt them to reflect as well as shape our musical environment. The most constant principle may well be the role performance traditions play in keeping interpretations connected within a musical community, that is, performances cannot be entirely subjective because they form part of a continuing dialogue in which they are both a cumulation of earlier performances and a catalyst for future ones (Hastings, 2006, p. 42).

These standards, which constrain music interpretation, are central to a more general definition of creativity as the “interplay between ability and process by which an individual or a group produces an outcome or product that is both novel and useful as defined within some social context” (Plucker et al., 2004, p. 156). Musicians must play what is written in the score and respect both stylistic norms and instrument-associated norms. Yet to offer an expert, artistic interpretation, the musician must also strive for novelty and originality in his or her musical interpretation. Thus, creativity in music interpretation may be described as the ability to follow musical and social norms while still proposing an original interpretation: to combine the expected and the unexpected. Creativity in music interpretation is an interplay between ability and the process by which a musician produces an interpretation that is relevant both to the written score (what to play) and to oral tradition (how to play), but is also recognized as original by both the general public and the musical connoisseur. For Brenneis (1990), from a socio-musicological point of view, the act of creating meaning from the abstract musical symbols written in the score with phrasing, dynamics, etc. is the creative activity in music performance. Clarke (2012) suggests that musical expression, which aims to communicate the structure of a piece and emotions through various cues, including tempo, articulation, tone, dynamics, and timbral effects such as vibrato (Juslin and Lindström, 2016), might be the key to interpretative creativity. Numerous studies analyze recordings of a single musical work performed by different musicians (Clarke, 1995; Cook, 2007b, 2008; Spiro et al., 2010), or multiple performances of a given work by a single artist (Chaffin, 2007), to show significant variations in the output.

How is it that interpretive results differ so significantly between musicians using the same symbols written in a score? What is happening on a day-to-day basis in individual practice sessions? The literature suggests that a musician must develop a mental representation of the piece he or she is working on (Lehman and Ericsson, 1997; Chaffin et al., 2003). Based on this mental image, the musician chooses (1) whether or not to enhance certain aspects of the structure of the music, such as a modulation or repetition, and (2) how to communicate emotion through tempo, articulation, tone, dynamics, and timbral effects (Juslin and Sloboda, 2010). Héroux and Fortier (2014) distinguish two aspects of the mental representation of a piece: first, the formal image, or what is written in the score, e.g., notes, rhythms, formal sections, tonality, etc., and second, the artistic image, or what is not written in the score, e.g., how the musician believes this music should sound. For Chaffin et al. (2003), the mental representation is the “mental map of a piece” that will allow the musician to determine performance cues he or she will use as “landmarks” to organize the work, memorize it, and stay concentrated during performance. Those cues may consist of basic elements such as fingerings, technical issues, or patterns. Performance cues may also be interpretative, i.e., the performer's musical choices, or expressive, i.e., intended to enhance a particular emotion or mood.

Chaffin et al. (2003) argue that mental representations guide individual practice sessions of a work according to a four-stage process. In the first stage, *scouting-it-out*, the performer reads the piece slowly in order to develop a mental representation of it and to identify structural and technical difficulties. In the second stage, *section-by-section*, the performer develops the motor skills to play the piece and makes aesthetic choices. In the third, the *gray stage*, the performer works through larger sections in order to automate his or her movements and to memorize the piece if necessary. In the fourth stage, *maintenance*, the performer consolidates his or her knowledge and focuses on final details in preparation for a public performance. To these four stages, Héroux and Fortier (2014) add an *artistic appropriation phase* during which the musician develops an artistic image of the work and seeks to express that image via an exploration of musical character, dynamics, tone, and phrasing. In this stage, the musician seeks a feeling of expressive precision and balance in his or her playing. In a previous paper that presented partial results of this research project (Héroux, 2016), the author described two musicians' differing approaches to the artistic appropriation phase. One musician, S3, sought to adhere to the score as much as possible, with the goal of conveying the internal message of the music via a formalist approach, while for the other musician, S2, it was important to convey a personal message. S2 used memories and a fictional narrative, which the author called “extramusical elements,” to help him to play with expression, thus taking a referentialist approach (Meyer, 1956).

This article describes a study investigating how nine expert musicians worked through the interpretation of an unfamiliar musical work for an audio recording. The researchers observed and documented the nine musicians during individual practice sessions and conducted follow-up interviews. A data set was collected from these different sources and then cross-referenced and validated with each participant in order to gain a more complete understanding of what had occurred in the practice room for each participant. This phenomenological approach makes it possible to investigate in depth the intimacy of cognitive creative processes encountered in the shaping of music interpretation. These processes are difficult to observe from outside because they rely on the musicians' experience and on their subjectivity. The aim of this research is twofold: to uncover the creative process underlying the shaping of an original interpretation and to develop a model to illustrate that process.

## Materials and methods

This study presents, on the one hand, a phenomenological point of view on each participant's experience in shaping interpretation. This approach allows us to enter into the intimacy of the creative act in the practice room and uses verbalizations, recordings, and interviews to access the participants' personal thoughts, emotions, memories, sensibility, and artistic process. On the other hand, this study also offers an overview of all of the participants' creative processes, including work stages, artistic appropriation, general and specific strategies, and cognitive processes.

### Contents of the individual practice session

We used the methodology of *Analyse par théorisation ancrée* (Paillé, 1994), which draws on the principles of Grounded Theory and provides for the analysis of several data types in order to understand a phenomenon and theorize it. This qualitative analytic method consists of a six-step approach leading to a gradual theorization of a phenomenon and is an act of conceptualization rather than the analysis of contents (Paillé, 1994). This iterative process involves the following six steps: *coding*, or labeling all the elements present in the initial corpus; *categorization*, or naming and conceptualizing the most important aspects of the phenomenon under study; *linking*, or analysis between categories; *integration*, or identifying the phenomenon's essential elements and then eliminating or creating categories; *modeling*, or attempting to reproduce the dynamics of the analyzed phenomenon; and *theorization*, or a meticulous and exhaustive construction of the “multidimensionality” and “multicausality” of the phenomenon under study.

The methodology used in this study was first presented in a pilot study (Héroux and Fortier, 2014), and then described in a publication that presented partial results from this research (Héroux, 2016). We chose nine expert classical guitarists, all of whom have completed either a master's degree (n: 5) or a doctorate (n: 4) in music performance at a university and/or conservatory in North America and/or Europe. When this study began, each had between 15 and 40 years of professional concert experience. The musicians were all instructed to practice the musical work *Why* (1987), from the cycle *Kinderlight* by American composer Andrew York (York, 1992). They began by sight-reading the piece and practiced until they judged themselves ready to record a final version in a studio context. The music presents no particular technical difficulties but is complex enough to require the work stages previously identified in the literature (Chaffin et al., 2003), although its length, 34 bars, minimizes the rehearsal time required to master the piece. None of the nine participants knew the piece beforehand, and they were not allowed to listen to it (either on recordings or on the Internet). Through this methodology, we sought to witness and document the process by which a musician makes a mental image of a piece and shapes an interpretation.

We collected and analyzed data using a variety of methods in order to gather information on (1) what happened during the musicians' individual practice sessions of *Why*, (2) creative moments experienced with another musical work, to be used as a basis of comparison, and (3) the musicians' musical values, their approaches to working on expressiveness in a musical interpretation, their musical working habits and strategies, and their thoughts on professional life and teaching.

The first round of data collection examined the musicians' individual practice sessions of *Why*, using methodologies intended for the observation of individual practice session processes (Nielsen, 2001; Chaffin and Imreh, 2002; Hultberg, 2008; Lisboa et al., 2011). We videotaped the individual practice sessions of the nine musicians, from their first sight-reading of the piece to a final recorded version, and instructed them to verbalize, in real time, their actions and thoughts during these practice sessions. Since the musicians may have forgotten to speak aloud at times, or may not always have been fully conscious of their actions (Chaffin and Imreh, 2002; Williamon et al., 2002; Theureau and Donin, 2005), this phase of data collection also included descriptions of the videotaped musicians' actions by a third-party observer other than the researcher. In addition, the nine musicians answered a self-reflection questionnaire regarding (1) any thoughts they may have had about *Why* between practice sessions, (2) the specific focus of each practice session, to be compared with their verbalizations during that practice session and with the third-party observer analyses of audiovisual recordings, and (3) moments when the participant felt particularly inspired or lost the notion of time. Tracking these moments allowed us to target important moments in the creative process in relation to cues characteristic of optimal experience or flow (Csikszentmihalyi, 1990). The questionnaire contained the following questions:

1. Since the last practice, have you thought about the musical work or performed any actions related to it?
2. Did you feel inspired?
3. Did you feel concentrated?
4. Did you feel effective?
5. Did you lose the notion of working time during this practice session?

After transcribing the participants' verbalizations during individual practice sessions and examining their answers on the self-reflection questionnaire, we identified practice moments for which we could not be sure what had occurred (e.g., no verbalization). To gather the missing information, we used a recall, or self-confrontation, interview (Theureau, 2010), with the recording of the practice sessions of *Why*. This technique has been used in research on professional expertise in various domains (Tochon, 1996; Trudel et al., 1996; Clot, 2004; Yvon and Garon, 2006), including music, where it has been used to study the activity of musical composition (Theureau and Donin, 2005).

### Interviews

The second round of data collection sought to gather information about the creative process of musical interpretation through an investigation of creative moments experienced while working on musical works other than *Why*. We asked our nine participants to recall one or two situations in which they had experienced very creative or inspiring moments, in order to determine in what forms and at what times such moments occurred. To do this, we used explicitation interviews (Vermersch, 2010),^3^ which enable the *a posteriori* verbalization of unconscious actions. This technique, focusing on the detailed description of an action, is used to identify unconscious but important professional knowledge (Bénetière, 1997; Vermersch, 2012).

The third and final round of data collection solicited information from the musicians regarding their musical values, approaches to conceptualizing interpretive expressiveness and musical creativity, and thoughts on professional life and teaching. We used semi-structured interviews with the following open-ended questions:

1. Was the way you worked on *Why* different from what you usually do? Please specify.
2. What is a quality interpretation?
3. What is an original interpretation?
4. What are the criteria that make an interpretation accurate and appropriate?
5. What is expressivity? What is its aim?
6. What is your conception of a performer's freedom in relation to score and style?
7. What is authenticity in music for you? What is valued?
8. In general, what kind of strategies do you use to work on music interpretation?
9. How do you teach what is a good interpretation? (discussion, exercises, requests, expectations, other)
10. When you play in concert, what do you try to do with the music? The audience?
11. How do you choose which music you will work on?

Transcriptions of all interviews were sent to the musicians for validation.

### Analysis

This study included two levels of data analysis: (1) each musician individually for an in-depth portrait of that musician's creative process; and (2) all musicians collectively for an overview of the creative process.

In order to proceed with content analysis of the individual practice sessions, the videos of these sessions were transferred as sources in nine separate “cases” in NVivo 8^4^. We transcribed (1) verbalizations made by the participants during individual practice sessions, and (2) additional information acquired in the recall interview. Actions identified by the external observer were coded in NVivo 8 as different layers of explanation over the video.

For each of the nine cases, we coded and analyzed the practice session data in order to identify four work phases (Chaffin et al., 2003) and the artistic appropriation phase (Héroux and Fortier, 2014). Following Chaffin et al. (2003), we coded practice sessions into four main categories based on the length of the passages worked on: short passages (up to 4 bars), medium passages (5 to 14 bars), long passages (15–34 bars), and complete chaining (34 bars). Subsequently, we identified and coded the actions carried out by the participant and grouped them by themes: reading, analysis of structure, choice, evaluation, assimilation, memorization, and visualization. These themes are associated with more specific work elements, such as nuance, attack, fingering, sound, motor gesture, voice discrimination, phrasing, tempo, expressivity, and use of extramusical inspiration. The identification of work elements and the length of the passages played allowed us to align our participants' processes with the work stages of Chaffin et al. (2003) and with the artistic appropriation phase (Héroux, 2016). To facilitate similar analyses of the contents of all of the practice sessions, we divided them into segments of about 10 min. When choosing where to begin and end these segments, we sought to avoid splitting a musical phrase or a participant's comments; as a result, the segments are slightly asymmetric in length, the shortest being 7 min and the longest 12 min.

Following Vermersch (2010), we used explicitation interviews to better understand the data collected during the practice sessions. We began by asking musicians to verbally “reconstruct” actions that they had carried out at specific moments. The researchers then organized these actions temporally. Content analysis (Bardin, 2006) was used to analyze data from the semi-structured interviews.

We then proceeded to *Analyse par théorisation ancrée*. This differs from content analysis (Bardin, 2006) in that it is not about counting recurrences and classifying a given content, but rather understanding and conceptualizing the content of a data set in order to explain a phenomenon (Paillé, 1994). *Analyse par théorisation ancrée* uses conceptualizing categories to “bring the analysis to the level of the understanding of a behavior, a phenomenon, an event or an element of a psychological or social universe” (Paillé, 1994, p. 160) and consists of six steps, as follows:

***Coding:*** In this study, we cross-referenced previously coded data from the individual practice sessions of *Why*, including participants' verbalizations, actions identified by the third-party observer, and information garnered via the self-confrontation interviews. We also coded data from the explicitation interviews regarding specific moments of creativity during the learning process for pieces other than *Why*, and from the semi-structured interviews regarding musical values, approaches to conceptualizing interpretive expressiveness and musical creativity, and thoughts on professional life and teaching.

***Categorization:*** We determined conceptualizing categories by grouping the coded data according to the wider phenomena to which they point. This process led us to subdivide certain conceptually dense categories, such as “Feeling the accuracy of expression in the playing.” We created a definition for each category and noted its properties, as suggested by Paill and Mucchielli (2008). This approach allows for ongoing validation of the data by the participants, either during subsequent observations or by direct questioning: “Does the analysis make sense? Do I understand the phenomena identified?”

***Linking:*** We determined (a) relationships between the conceptualizing categories (“empirical approach”) and (b) relationships between the conceptualizing categories and concepts in the literature (“theoretical approach”).

***Integration:*** We drew on the individual musical portraits that we had compiled of the nine participants in order to link the conceptualizing categories and integrate them into a larger structure.

***Modeling:*** We determined the structural and functional relations between elements (learning stages, actions, strategies, cognitive processes) characterizing the process of shaping an interpretation.

***Theorization:*** In this last step, we sought to explain the creative process via a visual representation (**Figure 4**) and accompanying discussion (below).

## Results and discussion

We have combined the results and the discussion sections of this article in order to better reflect the qualitative nature of this study. More information regarding the practice sessions, including the length of the excerpts practiced and the types of actions that the musicians performed, may be found in the Supplementary Materials. The Supplementary Materials also include detailed descriptions of the work phases and the artistic appropriation phase for each musician. In addition, the nine musicians' studio recordings of *Why* may be found at https://youtu.be/AS0SiLHpo2g. These recordings aurally illustrate some elements of the following discussion. However, we do not offer an analysis of these recordings, as they were merely the pretext that allowed us to study the creative process during musicians' practice sessions.

### Participant information, experiment duration, and self-reflection questionnaire answers

The first column of Table 1 contains participant identification codes. The second column shows the number of years of professional experience for each participant and his or her diploma, where M = master's degree and D = doctorate or equivalent, e.g., Conservatory First Prize. The third column shows the number of practice sessions, the total number of minutes, and the total number of weeks for each participant from the first sight-reading of *Why* to the final recording. The remaining columns identify those practice sessions for which the participant answered “yes” to a given question on the self-reflection questionnaire. In these columns, “na” means “no answer,” which we consider to be different than a “no” response. Specifically, S1 forgot to answer Q5 in R2, and S2 forgot to answer the whole questionnaire in R3 and R4. S9 did not record the last practice session (R3) or answer the self-reflection questionnaire for these practice sessions; he thought it was irrelevant, because these practice sessions consisted of mental/visualization practice.

**Table 1:** Participants and practice session information with self-reflection questionnaire (RQ) results.

**Participant identification**	**Years of experience M = Master's degree D = Doctorate**	**Total individual practice sessions**	**Minutes rehearsed**	**Weeks rehearsed**	**Q1 Since the last practice session, have you thought about the musical work? Auditive Reminiscences**	**Q2 In this practice session, did you feel inspired?**	**Q3 In this practice session, did you feel concentrated?**	**Q4 In this practice session, did you feel effective?**	**Q5 In this practice session, did you lose the notion of working time?**
S1	10 M	8	249	6	R: 1-2-3, 4	R: 1, 2, 3, 4, 5, 6, 7	R: 13, 4,5, 6,7	R: 1, 2, 3, 4, 5, 6, 7	R1, 3
S2	25 M	4	105.20	4	R: 1-2-3-4	R: 1, 3 (R4na)	R: 1, 2 (R4na)	R: 1, 2, 3 (R4na)	R: 1, 2, 3 (R4na)
S3	40 D	2	103.42	2	0	R: 1, 2	R: 1, 2	NRP	R: 1, 2
S4	10 M	9	326.89	19	0	R: 2, 3, 4, 6, 7, 8	R: 2, 3,4, 6,7, 8	R: 3, 4,7, 8,	R: 2, 3, 4, 6, 7, 8
S5	35 M	4	72.96	20	R: 1-2	0	^1^R: 1, 2	R: 1, 2	R: 1
S6	40D	6	260.54	31	R: 2, 3, 4, 5, 6	R: 1, 2, 3, 4, 5, 6	R: (R2 na), 3, 4, 5, 6	R: 1, 2, 3, 4, 5, 6	R: 1, 3, 4, 5, 6
S7	15D	4	86.60	38	0	R: 1, 3	R: 1, 3, 4	R: 1, 2, 3, 4	R: 1, 3
S8	15 M	6	217.93	26	0	R: 1, 2, 6	R: 1, 2, 3	R: 1, 2,3, 6	0
S9	15D	3 (1 without guitar/no record)	64.58	4	(R3na)	R1, 2 (R3na)	R1, 2 (R3na)	R1, 2 (R3na)	(R3na)

Since we instructed the participants to choose the moment that they felt ready to record *Why*, the duration of the experiment varied from one participant to another. The number of practice sessions ranged from two to nine, and the number of weeks ranged from two to thirty-eight.

We hoped to use the first question (“Since the last rehearsal, have you thought about the musical work or performed any actions related to it?”) to collect information about auditory reminiscences (incubation) between practice sessions without asking directly about this topic. Therefore, we retained only answers related to auditory reminiscences and ignored other responses (e.g., “Yes, I did say to myself that I have to find time to practice the music”). Participants S1, S2, S5, and S6 mentioned auditory reminiscences. In response to the second question (“In this practice session, did you feel inspired?”), only S5 never mentioned feeling inspired. In response to the third and fourth questions (“In this practice session, did you feel concentrated” and “[…] did you feel effective?”), all participants felt that they were concentrated and effective most of the time. In response to the last question (“In this practice session, did you lose the notion of working time?”), S8 and S9 were the only participants who never answered yes.

### Work stages and artistic appropriation

The results of the content analysis for each participant's practice sessions, including length of excerpts practiced and types of actions performed, are presented in the Supplementary Materials under the heading *Contents of Individual Practice Sessions*. The following section summarizes these preliminary analyses, which we used to identify work stages and the artistic appropriation phase, as detailed in Table 2 below.

**Table 2:** Categories of strategies used.

**Use of musical structure (*n* = 9)**	**To develop artistic image**	**To play with expression**	**To “get in the mood”**
Analyze: form, harmony, shape of the melody, composer's notes (S1, S2, S3, S4, S5, S6)	x	x	
Use the rests in the score (S2, S3, S5, S9)		x	x
**USE OF MUSICOLOGICAL REFERENCES (*****n*** = **9)**			
Apply the subject's choices in regard to historical, cultural, and geographical notions (S2, S3)	x		
Compare the piece to other works by the composer or other works of a similar style (S3)	x		
Listen to and/or reference other performances of the piece - With the goal of imitation (S1, S2, S3, S6) - With the goal of playing the piece differently (S2, S5, S7)	x	x	
Visualize an orchestrated version of the piece and make expressive associations based on such a version (S1)	x	x	
Make references to other pieces that the participant has already worked on (S2, S3, S6, S9)	x		
**EXPLORATION (*****n*** = **9)**			
Explore different musical ideas with expressive “tools”: fingering, rhythmic feel, length of held notes, articulation, changing dynamics, timbres and tempos (S1, S2, S3, S4, S5, S6, S7, S8, S9)	x	x	
Play voices individually (S2, S5, S7)	x	x	
Rethink how the piece will be played/Play differently (S1)		x	
Transfer the work done on another instrument (S6)	x	x	
Improvise on the music (S1, S8)	x	x	
Play with the final version in mind from the start (S1, S2)		x	x
Play the same passage with different performance traits (S4, S5)	x	x	
Play the piece slowly with the aid of a metronome to recover musical and physical comfort (S3)			x
Master a neutral version, notes and rhythm in time (S1, S6)	x		
**EVALUATION (*****n*** = **9)**			
Record own work (S9)	x	x	
Evaluate the coherence between the **score** and the musical version produced (S1, S2, S3, S4 S5, S6, S7, S8, S9)	x	x	
Evaluate the coherence between the **mental** version and the musical version produced (S1, S2, S3, S4, S5, S6, S7, S8, S9)	x	x	
Perform the piece in front of an audience (S4)	x	x	
**USE OF EXTRA-MUSICAL SUPPORTS (*****n*** = **7)**			
Write down key words/images to draw out certain sensations/feelings (S3, S4)	x	x	x
Stimulate emotions through memories (S2, S3, S6, S9)	x	x	x
Look for physical sensations (gesture ease, relaxation) to help performance (S2, S3, S4, S5, S6, S9)	x	x	x
Use analogies with musical material (S8, S9)	x	x	x
Develop a narrative or story based on the music (S2)	x	x	
Associate colors to the parts of the piece's harmony (S2)	x	x	
**PSYCHOLOGICAL STRATEGIES (*****n*** = **6)**			
Follow one's instinct (S1)		x	X
Feel the music (S1)		x	X
Find ways to focus: detachment from one's ego, letting go, connecting with one's inner self, etc. (S1, S2, S3, S8, S9)		x	X
Avoid too much emotional investment (S3)			X
**MEMORIZATION (*****n*** = **5)**			
Try playing the piece by heart (S4)	x		
Memorize physical sensations (S4, S6)		x	X
Write down musical ideas (S4, S2, S3) or a formal analysis (S1, S3, S6) on the score			X
**VISUALIZATION (*****n*** = **4)**			
Work on the piece mentally between practice sessions with or without the score (S2, S9)	x		
Hear the piece while reading the score (S6)	x		x
**INCUBATION (*****n*** = **5)**			
Take a break or work on another piece (S7, S8)	x		
Give the brain time between practice sessions to work on the piece subconsciously (S9)			
Hear the piece in one's head (S2, S6, S7)	x		
**OTHER (*****n*** = **2)**			
Erase or ignore expressive indications written in the score (S1, S9)	x	x	

#### S1: eight individual practice sessions

We divided the first practice sessions into three segments. We identified a short scouting-it-out phase on the first of these segments. On the second segment, the participant worked on short and very short passages and subdivided the work into sections. This section-by-section phase continued until the end of the second practice session, when the first detachments from the score took place. The participant entered a long gray stage from sessions three through six. During this stage, we noticed a focus on memorization and a progressive detachment from the score. We classified the last two practice sessions under the maintenance stage.

As of the seventh practice session, the participant was finished with memorization and was working on the piece as a whole. On the last segment of the last practice session, we observed a return to working on short passages and memorization. We considered these to be final revisions leading up to the final recording. It was difficult to identify the artistic appropriation phase in this participant's practice, since he worked on it throughout the practice sessions. The participant's inspiration was notable throughout his practice as well. Furthermore, the participant did not use extramusical elements to support his artistic appropriation.

#### S2: four individual practice sessions

For participant S2, the scouting-it-out phase took place during the two first segments (out of four) in the first practice session. During this time, the participant worked on elements linked to this phase, including run-throughs and a preliminary analysis of the piece. The third segment of the first practice session marked the beginning of the section-by-section stage, which continued until the first segment of the second practice session. At that point, the participant often returned to reading the piece throughout, but analyzed it less frequently. During the section-by-section stage, the participant made many choices concerning fingering and technique. Moreover, the participant worked on memorization from the first practice session through the first segment of the second practice session, when he was in the section-by-section stage and focused on short sections of the piece. The gray stage began with the second and last segments of the second practice session and ended at the end of the third practice session. From this stage on, the participant began to work on playing the entire piece while retaining the musical choices made in the section-by-section stage. Meanwhile, elements linked to the second phase, such as fingering, technique, and expressive choices, progressively diminished until the end of this phase. The last practice session was associated with the maintenance stage. During this stage, the participant repeated the piece many times and focused on technical aspects, with the goal of generating a consistent end product. Elements related to the artistic appropriation phase were present throughout S2's process, except during the last segment of the third practice session and the first segment of the fourth. These elements returned in the very last segment, before the participant began to record.

#### S3: two individual practice sessions

The work stages and the artistic appropriation phase were hard to distinguish for S3. The scouting-it-out stage seemed to be limited to the first segment of the first practice session. In this segment, the participant read through the piece three times and did a substantial musical analysis of the piece. This musical and stylistic analysis of the piece continued throughout the practice session, though less frequently. We also noticed a few analyses over the course of the second practice session. The section-by-section stage began on the second segment of the first practice session. In fact, S3 worked on small sections of the piece throughout the first two practice sessions, except in the third segment of the second practice session. However, we allocated the whole of the second practice session to the gray stage because, during this practice session, the participant worked on achieving a rendition of the piece in its entirety. It should be noted that we didn't identify any detachment from the score from S3. His work never reached the maintenance stage since he didn't make any creative choices until the very end of the second practice session. We noticed some factors associated with musical appropriation in the last segment of the first practice session and the third segment of the second practice session.

#### S4: nine individual practice sessions

For S4, the scouting-it-out phase was present throughout the first two individual practice sessions. From the third practice session on, we noticed fewer elements linked to this phase and more work on short and very short sections of the piece. Therefore, we considered that the section-by-section stage began with the third practice session and ended in the penultimate segment of the fourth practice session. From the last segment of the fourth practice session to the fourth segment of the eighth practice session, we noticed a significant amount of time dedicated to the gray stage. During this period, the participant began playing the piece from beginning to end and memorizing it. Throughout this process, however, we observed a regression toward working on short passages, and read-throughs of the piece following a break. In the last segment of the eighth practice session, the participant entered the maintenance stage. The participant continued to work on memorization during the eighth practice session but felt that her phrasings were “where they need[ed] to be” (QR.Q2.R8). During the last practice session, the participant practiced playing the piece from beginning to end to prepare for recording. The artistic appropriation phase was difficult to distinguish in the data. The participant sometimes used outside musical support but confirmed in an interview that this was necessary for her only in the context of this project. The participant also confirmed that she developed the musical interpretation very little during her work on *Why*.

#### S5: four individual practice sessions

For S5, it was difficult to distinguish work stages based on length of excerpts practiced because the percentage of time dedicated to short, medium-length, and long passages, respectively, remained fairly constant throughout the individual practice sessions. He spent 80–95% of each practice session working on short and very short passages, 4–15% working on medium-length passages, and 1–7% working on longer passages. Maintenance was the only discernible stage during the last practice session. To distinguish the different stages, we focused only on what the participant chose to work on and not on the length of the excerpts practiced. The scouting-it-out stage was restricted to the first two segments of the first practice session, when we observed two read-throughs of the piece and some analysis. For the remainder of the first practice session, the participant seemed to have entered the section-by-section stage. He made many decisions (fingering, technical, musical) and although he began trying to play the piece from beginning to end, small passages dominated the practice session. From the second practice session onwards, the participant began working on memorization and continued to attempt complete renditions of the piece. The participant then entered the gray stage, which continued until the end of the third practice session. During the last practice session, the participant did not make any new decisions regarding performance, but rather worked on playing the piece in its entirety to prepare for the upcoming recording. We considered this to be the maintenance stage. It was difficult to pinpoint the artistic appropriation phase in the data we collected. The musical interpretation work was constantly present, and the participant used only one source of outside musical support during the individual practice sessions.

#### S6: six individual practice sessions

The scouting-it-out phase took place during the first practice session for S6. From the start of the second practice session through the third segment of the third practice session, the participant entered the section-by-section stage. The participant worked primarily on short passages until the end of the second practice session, when the participant worked through several consecutive long passages. In the penultimate segment of the third practice session, the participant began to work on the piece as a whole. The participant played more and more medium-length passages, marking the gray stage. Finally, at the end of the fifth practice session, a complete rendition of the piece marked the beginning of the maintenance stage. Though the participant revisited certain performance decisions during the sixth practice session, this was in preparation for recording and therefore still in the maintenance stage. The first two segments from the final practice session were mainly dedicated to performance and included complete renditions of the piece.

#### S7: four individual practice sessions

Participant S7 seemed to become familiar with the piece very quickly. In the first reading, he completed many medium-length and long passages. In the scouting-it-out stage, during the first segment of the first practice session, the participant not only read through the piece but also analyzed it. From the second segment of the first practice session through the end of the second practice session, the participant was in the section-by-section stage. The participant continued to analyze the piece while also focusing on fingering, performance, and technical aspects of the piece. The participant continued to work on short and very short passages throughout the remaining practice sessions. However, from the third practice session onwards, the participant began working on a complete rendition of the piece. He had therefore entered the gray stage. Only on the last segment of the fourth practice session did we see indications that the participant had entered the maintenance stage, when he played the piece in its entirety in anticipation of the upcoming recording. Note that the participant also made performance decisions during this segment.

#### S8: six individual practice sessions

For S8, the scouting-it-out phase took place over the four first segments of the first practice session, when the participant conducted some read-throughs and analyzed the piece extensively. In the fifth and last segment of the first practice session, analysis became less of a priority, and the work on short and very short sections took precedence 98.65% of the time. We therefore selected this moment as the beginning of the section-by-section stage. This stage continued until the end of the fourth practice session. Note, however, that the participant worked on short and very short passages and made many performance decisions throughout the practice sessions. At the end of the fifth practice session, the participant began the gray stage. During this practice session, the work on short and very short passages declined significantly to make way for work on longer passages. Moreover, we noticed many detachments from the score. During the sixth and final practice session, the participant was in the maintenance stage, making no changes and playing the piece from beginning to end twice.

#### S9: four individual practice sessions

S9 did not rehearse many times and did not record the last two practice sessions, when he only visualized the piece without an instrument. The scouting-it-out stage took place during the first segment of the first practice session. The participant looked over the piece without playing it, played it through a few times, and analyzed it. The section-by-section stage began with the second segment of the first practice session. With the exception of the last segment of the first practice session, when the participant attempted to play the piece from beginning to end, short and very short sections made up between 93 and 96% of the participant's work. Although the participant did not record the last practice session, he confirmed that the third practice session was entirely consecrated to memorizing the piece. We characterized this as the gray stage. The participant confirmed that the fourth and final practice session was used to “consolidate mental images associated to the piece” and “validat[e] memorization through visualization” (S9.R03.2015.02.22). Since this practice session consisted of many elements used to prepare for the upcoming recording, we classified this as the maintenance stage.

#### Work stages and artistic appropriation as part of an interactive process

We were able to identify work stages for most of our participants, but our data shows that the boundaries between these stages were not well defined. We observed participants moving back and forth between different stages. For example, in the gray stage, S4 also read through and worked on short passages. This may be explained by the participant's return from a practice session break lasting several weeks, or simply by his need to return to an earlier stage of practice. In the maintenance stage, S7 made performance decisions that are more closely linked to the scouting-it-out phase. In addition, we were not able to clearly identify the steps for S3 and S5. Wise et al. have similarly argued that shaping an interpretation “is not as linear as Chaffin suggests in his four stages” and their data, like our own, “challenges any notion that a given performance is an end point or that an interpretation is ‘finished’ and final” (2017, p. 157).

Most of our participants settled temporarily on an artistic image of the piece in order to prepare for performance, though S3 and S5 continued to change it on the spot; according to S3, “it is never the same, never” (S3.EDS.113).^5^ All participants told us that their artistic image of the music changes both after a performance and over time, whether or not they continue to play the piece, because “our knowledge [and] our way of seeing things evolve”^6^ (S3.EDS.115). The interactive process model of Wise et al. (2017) reflects our observations, and its three components are similar to concepts described elsewhere in the literature. “Developing an overarching concept” is quite similar to the concept of making a mental representation of the music (Sloboda, 1985; Lehman and Ericsson, 1997; Chaffin et al., 2003); “Establishing focused intentions” may relate to performance cues (Chaffin and Imreh, 2002); and “Making it feel right” suggests the artistic appropriation stage (Héroux and Fortier, 2014), wherein the musician seeks accuracy in the interpretation.

To understand the process of artistic appropriation as a whole, we used both the recordings of the individual practice sessions and data from the interviews. For the majority of participants, artistic appropriation was continuous from the scouting-it-out stage to the performance. S2 told us that the appropriation stage usually begins before the first reading of the music, when he listens to recordings and tries to find a personal message or storyline he can express within the music: “For me it's a lot nicer to play these things when I have […] my storyline, when I have a REASON to play”^7^ (S2.ESDb.8). This approach was not possible in this experiment, as we instructed the musicians not to listen to other interpretations of the work (Héroux, 2016). For S1, S4, and S8, information in the interviews explained why the artistic appropriation stage may not have been clearly observable during the recorded individual practice sessions. S4 needed to memorize the music before artistic appropriation began: “to know it by heart, first.” S1 and S8 stated that it was only when they played the piece or recorded it on the spot that they reached this phase: “That's after […] it's a step later. When I'm in the studio”^8^ (S8.EE.46:44).

### Strategies used

Strategies used are presented in Table 3, Annex 2. In the first column, the strategies identified are grouped in eight categories: Use of musical structure, Use of musicological references, Use of extra-musical support, Exploration, Visualization, Incubation, Evaluation, and Other. This column also identifies the strategies used by each participant. Columns two, three, and four show the purposes of each strategy: (1) to develop an artistic image, i.e., to help the participant gain a clearer idea of how the music should sound; (2) to play with expression, i.e., to improve the participant's ability to realize, through the instrument, his or her artistic image of the piece; and (3) to “get in the mood,” i.e., to help the participant find the right mental and physical disposition to play.

#### Musical structure and musicological references

All musicians used both an analysis of the musical structure of the piece and musicological references to develop a mental image and play with expression. They made references to other pieces they had previously worked on (S2, S3, S6, S9), compared the piece to other works by the composer or other works of a similar style (S3), and made expressive associations based on their visualization of an orchestrated version of the piece (S1). Some participants mentioned using the rests in the score to enhance the expressivity of the musical structure and to evoke mood changes in the music; as stated by S3, “In this silence, there is the physical and mental preparation to the next event, the next note”^9^ (S3.EE. 28:04). Participants also used strategies to evaluate the coherence between their mental image, their playing, and the written music. One musician recorded his playing (S9) and one performed the piece in front of an audience (S4).

#### Exploration

All participants used a variety of strategies to develop their mental image of the piece, play with expression, and, to a lesser extent, “get in the mood” for playing. One strategy common to all musicians was to explore various expressive “tools”: fingering, rhythmic feel, length of held notes, articulation, changing dynamics, timbres, and tempos. Other, less common strategies included: playing voices individually (S2, S5, S7); rethinking how the piece might be played differently (S1); transferring work done on another instrument to the guitar (S6); improvising on the music (S1, S8); playing with the final version in mind from the start (S1, S2); playing the same passage with different performance traits (S4, S5); playing the piece slowly with the aid of a metronome to gain musical and physical comfort (S3); and mastering a neutral version with the notes and rhythm in time (S1, S6).

#### Extramusical supports

This category includes strategies related neither to musical structure nor to musicological elements. Seven of the nine participants used extramusical supports. Strategies included looking for physical sensations (gestural ease, relaxation) to help performance (S2, S3, S4, S5, S6, S9); stimulating emotions through memories (S2, S3, S6, S9); writing down key words or images to draw out certain sensations or sentiments (S3, S4); making analogies between the musical material and extramusical concepts (S8, S9); developing a narrative or a story based on the music (S2); and associating colors to parts of the piece's harmony (S2).

The most explicit example of an extramusical support, a narrative, was given by S2 in a recall interview while watching the recording of his first practice session. For him, the music illustrates a man and a woman talking while going for a walk in November, when leaves are falling on the already leaf-covered ground. He imagined their conversation as easy at the beginning, but increasingly confrontational as the musical harmony intensifies. Parts of this narrative corresponded to specific musical details and at times he altered his guitar technique to fit the musical idea generated by this narrative:

The man thought it was okay [what he said], then the woman said, ‘No, no, no.’ […] He tried to say it again… Then there, she got upset… She shouted—but already I knew she should not shout, my glissando was too aggressive for the message that I knew I wanted to say. […]. He [the man] said: “Oh, uh… I did not know.” Then he understood [her point]. Harmonics [written in the score], for me, it's the conversation that stops, and then they think. It's like little bubbles that we see in comics^10^. (S2.EA. P11)

#### Psychological strategies

This category includes strategies used by six of the participants to “play with expression” and “get in the mood.” The most frequent strategies were those used to increase focus: detachment from one's ego, letting go, connecting with one's inner self, etc. (S1, S2, S3, S8, S9). Additional strategies included following one's instinct (S1), feeling the music (S1), and avoiding too much emotional investment (S3). None of the participants seemed to use these strategies to develop their artistic image of the music.

#### Incubation

We observed that three of the participants spontaneously heard the music in their heads between practice sessions (S2, S6, S7). This may have helped to refine the artistic image. Additional strategies included giving the brain time between practice sessions to work on the piece subconsciously (S9) and taking a break or working on another piece (S7, S8).

#### Memorization

Although we didn't ask participants to memorize the music, we observed four memorization strategies used by five participants. These strategies helped the participants develop a mental image of the piece, play with expressivity, and get in the mood. This category also includes strategies related to annotations on the score, which five of the participants used to remember ideas and information from one practice session to the next. Strategies in this category include memorizing physical sensations (S4, S6); writing musical ideas on the score (S4, S2, S3); writing a formal analysis on the score (S1, S3, S6); and trying to play the piece from memory (S4).

#### Visualization

Visualization strategies were not used for memorization. Rather, participants used these strategies to work on the music without an instrument, to develop an artistic image of the piece, and to “get in the mood.” Strategies included hearing the piece while reading the score (S6), and working on the piece mentally with or without the score between practice sessions (S2, S9).

#### Other

We observed one strategy that did not fit into any of the above categories: erasing or ignoring expressive indications written in the score (S1, S9).

### The impact of context and personal style

**Figure 1:**
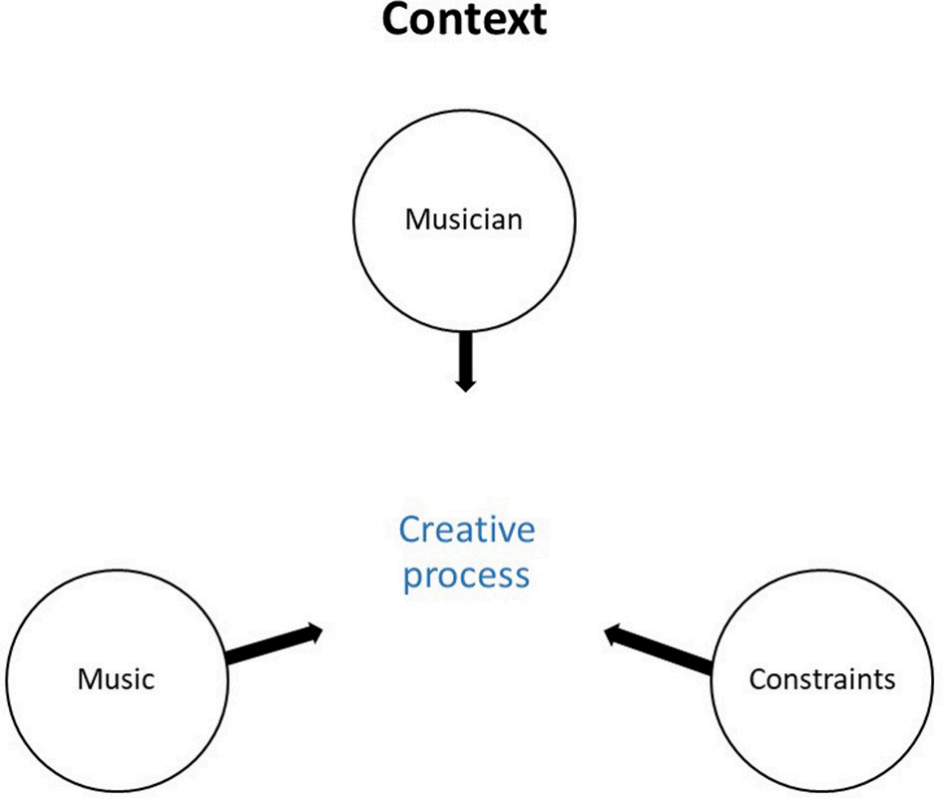
The context in which the creative process took place.

The role of the creative process in shaping an interpretation depends on contextual elements (Figure 1). Using the recordings of the individual practice sessions and the interviews, we were able to find elements related to the musicians (academic background, learning style, values), their professional constraints (deadlines, public), and the music (style, difficulty level) that explained the similarities and differences in our results.

#### Background and personality

All nine participants used strategies related to analysis of the musical structure and to musicological references in order to develop a mental image and to play with expression. This is not surprising, as these strategies are valued in the academic training of musicians: since the twentieth century, the tradition in classical music has been to follow the score and try to play in an appropriate style, according to “les règles de l'art,” in a balance between written and aural traditions (Hastings, 2006). As anticipated by the literature on the importance of mental images in practicing (Lehman and Ericsson, 1997; Chaffin et al., 2003; Sloboda, 2005), all of our participants used strategies to evaluate the coherence between their mental image, the results of their playing, and the written music. All participants also explored different musical ideas using expressive “tools.” This strategy reflects the ways in which they were trained to work on music.

If our participants' backgrounds may explain certain similar strategic choices, we observed other choices that were less common and may reflect individual learning styles, or “evidence of distinct analytic and intuitive approaches to interpretation” (Hallam, 1995, p. 117). Wise et al. (2017) describe such choices as either “musical parameter-led […] seeking balance between, and an effective understanding of, different musical elements in relation to the whole” (151), or “emotion/narrative-led […] seeking/communicating emotional narrative or effects” (153). For example, S3 described searching for the piece's “coherence” while S2 was definitively emotion/narrative-led: “I start to find the necessary tools at home to make me cry while also hoping the public will cry. […] I start to be moved by those things that I created there”^11^ (S2.EA. R4.15:40).

#### Values

Values offer another explanation for the musicians' choice of strategies, personal learning styles, and musical choices. Wise et al. (2017) note the “tension between, on one hand, the respect that performers often feel they must have for performance traditions and the score—as somehow enshrining the composer's intention—and, on the other hand, their own personality and individuality, factors which can be seen as essential to creative performing” (157).

Our participants had never heard *Why* before this study, so they were not influenced or inspired by another musician's interpretation. As expected, they all stated that they sought to express an overall atmosphere defined by the title, *Why*, and the composer's indication “with sorrow.” With respect to the latter, S1 stated, “Of course, the word ‘Sorrow’ points to […] what atmosphere there is, which emerges from this music, an atmosphere a little more… introspective… Maybe a little melancholy”^12^ (S1.EA.1.7:54). S3 was influenced by the combination of the two terms:

[T]he conjunction of 'why' and 'sorrow' is interesting. […] there is the aspect of sadness, but there is the aspect of questioning around it, so…It is peculiar to link the two concepts. Well, it can bring us back to the notion of statement […] It is like thoughts, a little scattered, in some way. And that goes with questioning. We often ask ourselves questions; we are a bit lost and we question something. We ask ourselves questions, but asking oneself questions doesn't necessarily mean having an absolutely straight path^13^. (S3.EA.7.36:14)

Some of our participants placed a higher value on what Taruskin (1995) identifies as authenticity in music: namely, the score and stylistic considerations. For example, according to S7, it is imperative to respect the indications of the musical text and the composer's intentions, and “to be at the service of the music as much as possible” ^14^ (S7.EDS. 38:37,00). He does not believe that a musician should place himself before the music, an approach that he describes as “narcissistic” (S7.ESD.163).

**Figure 2:**
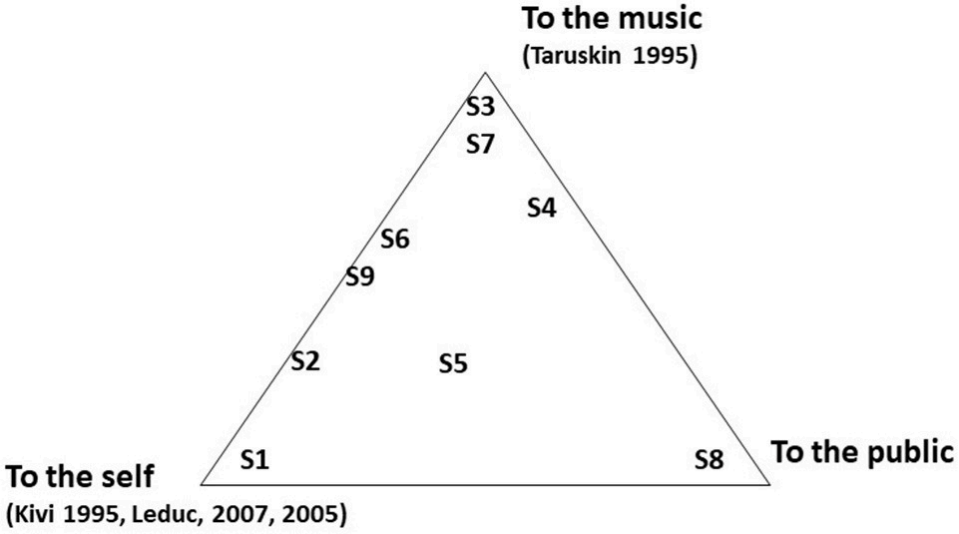
Type(s) of authenticity valued by the participants.

Other participants, by contrast, valued what Kivy (1995) and Leduc (2007) term “authenticity to the self,” i.e., how they feel about what they have to express and how to express it. S1 remarked, “I do what I want, and if I want to play it this way, I'll play it this way”^15^ (S1. ESDc: 11). Indeed, on S1's final recording of *Why*, he has chosen a surprisingly slow tempo with less rubato than the other participants, giving rise to a more meditative atmosphere. This unusual interpretation could be explained by the authenticity to the self this participant values: “[…] that's how, for me, I perceive it, what it makes me feel when I play it”^16^ (S1.EA.2.18.25). By contrast, the final recording by S6 stands out for his dynamic interpretation, faster tempo, and obvious rubato. While he also values the importance of putting a bit of himself into the music, he noted a difference between interpreting older music (e.g., Bach) and newer repertoire: “[W]hen one works from the repertoire of a well-known composer or period, one tries to capture the style or the authenticity of the style, of the piece […] for example, in Baroque music, one respects the processes of Baroque interpretation”^17^ (S6.EDS.10). Other participants also had nuanced responses about authenticity: S5 stated that, in a musical interpretation, “we see him [the musician] […] but we also see the music”^18^ (S5.EDS.27). A visual representation of the type(s) of authenticity valued by the participants is shown in Figure 2.

#### Professional constraints

According to S8, authenticity “depends on the audience”^19^ (S8.ESD.10). When performing for musicians, he follows standard stylistic and technical norms and adheres closely to the score, whereas he may offer a more personal interpretation in front of a non-expert audience. S8 usually performs in hospitals for patients in palliative care, playing repertoire requested by the patients themselves. S8 arranges and interprets the music with his public's anticipated reaction in mind. Given the constraints of this professional context, S8 must prepare pieces within a very short time frame.

We may also presume that the level of difficulty of the piece would affect the strategies used, though we were not able to observe this in our study.

### Cognitive processes

We did not directly observe cognitive processes, as we were not able to document activity in the brain. However, by identifying the results of mental operations and observing behaviors and strategies, we may better understand the processes by which musicians shape an interpretation and find originality.

#### Importance of alternating between divergent and convergent thinking

Our analysis of the data shows that the musicians used strategies related to musical structure and musicological references to help refine their mental image of the piece: “While sight-reading, I search for what [the piece] holds, in terms of musicological information, in order to interpret it”^20^ (S6.EE.1.16:09). The musicians in our study used exploration strategies and divergent thinking processes to generate new musical ideas and find originality in their interpretation of *Why*. For example, S6 changed accentuation and nuances in a random way: “In general, I would say that when I don't know what I want to do, or if I feel I'm playing mechanically, I'll start doing some things arbitrarily to rekindle my sense of spontaneity, you know”^21^ (S6.EE. 76.43:00). The musicians also used evaluation strategies and convergent thinking processes to validate the results of their experimentation with both the score and the mental image. These strategies allowed the musicians to ensure that their musical result was relevant to the context (style, score, etc.). “Because this brings me closer to what I hear in my head […] I feel nearer the music, closer to the idea that I have of the piece and the way that I performed it”^22^ (S5.EE.83.17:10). S7 described alternating between divergent and convergent thinking processes: “I play it, and if something comes to me spontaneously or intuitively, well then I will try to verify it [with the score]. Is there something that supports it? If so, well, then there it is, bingo. If not, well, then I'll continue to look”^23^ (S7.EA.5:10). In other words, he uses an analysis of the musical structure to validate, or invalidate, his spontaneous musical ideas.

**Figure 3:**
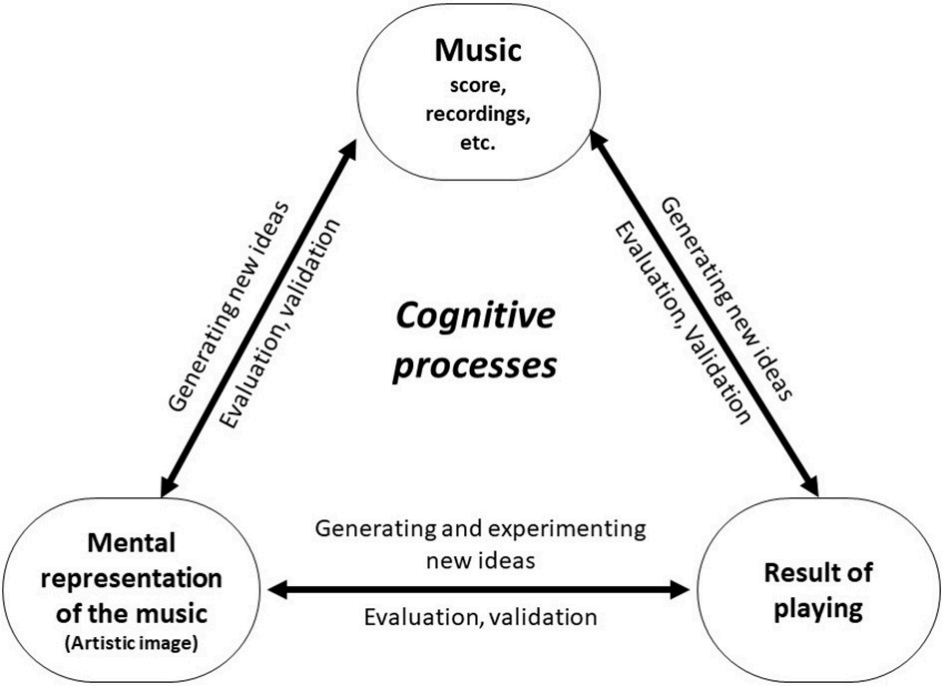
The three pillars of the creative process in shaping interpretation.

This dynamic of generating, testing, and validating ideas in practice sessions operates between three pillars: the music, the mental representation, and the results of playing (Figure 3). This constant back-and-forth between divergent and convergent thinking is the basis of the creative process (Guilford, 1968). In this experiment, we observed the basic mental cognitive process of creativity, the “interplay between ability and process by which an individual or a group produces an outcome or product that is both novel and useful as defined within some social context” (Plucker et al., 2004). We also observed two additional cognitive processes that were used as strategies: creative associations and incubation.

#### Creative association

To generate new musical ideas and explore expressivity, seven participants used extramusical supports. As described above, S2 used a narrative that followed the musical discourse: “So that's what I'm looking for here: will we get to the point where in my scenario, the woman is going to scream, or not? I'm playing with that”^24^(S2.EA.2. 10:00). Other musicians used memories or analogies to feel an emotion that would help them play a musical phrase with that emotion. As S6 explained, “I had to find a way to feel nostalgic like that, it's a very nostalgic piece. For me. And here I tried to remember what they do, the method actors, where they relive sad moments. And it helped me a lot”^25^ (S6.EE.119.1:04:23).

According to the Emotional Resonance Creativity Model (Lubart and Getz, 1997; Lubart, 2015), analogies and metaphors result from emotional or affective correspondences between a task (here, the work of interpretation) and emotions felt in the past in very different situations. For example, the title, *Why*, and the expressive indication, “With sorrow,” triggered in S3 a form of resonance that generated a creative association, as described above. S3 described how the composer's indications influenced his phrasing in the second practice session:

This phrase ending should always slow down, because it relates to the two words that are there, “sorrow” and “why.” So there is a questioning aspect in the act of slowing down, we are no longer certain. We utter something and after that, we are no longer sure. […] It's here that lies the ‘sorrow.’ It is coherent.^26^ (S3.R2:15)

Creative associations are important elements of the creative process (Lubart and Getz, 1997; Lubart, 2015); our participants used them as strategies to generate musical ideas or to help them play with expressivity.

#### Incubation

**Figure 4:**
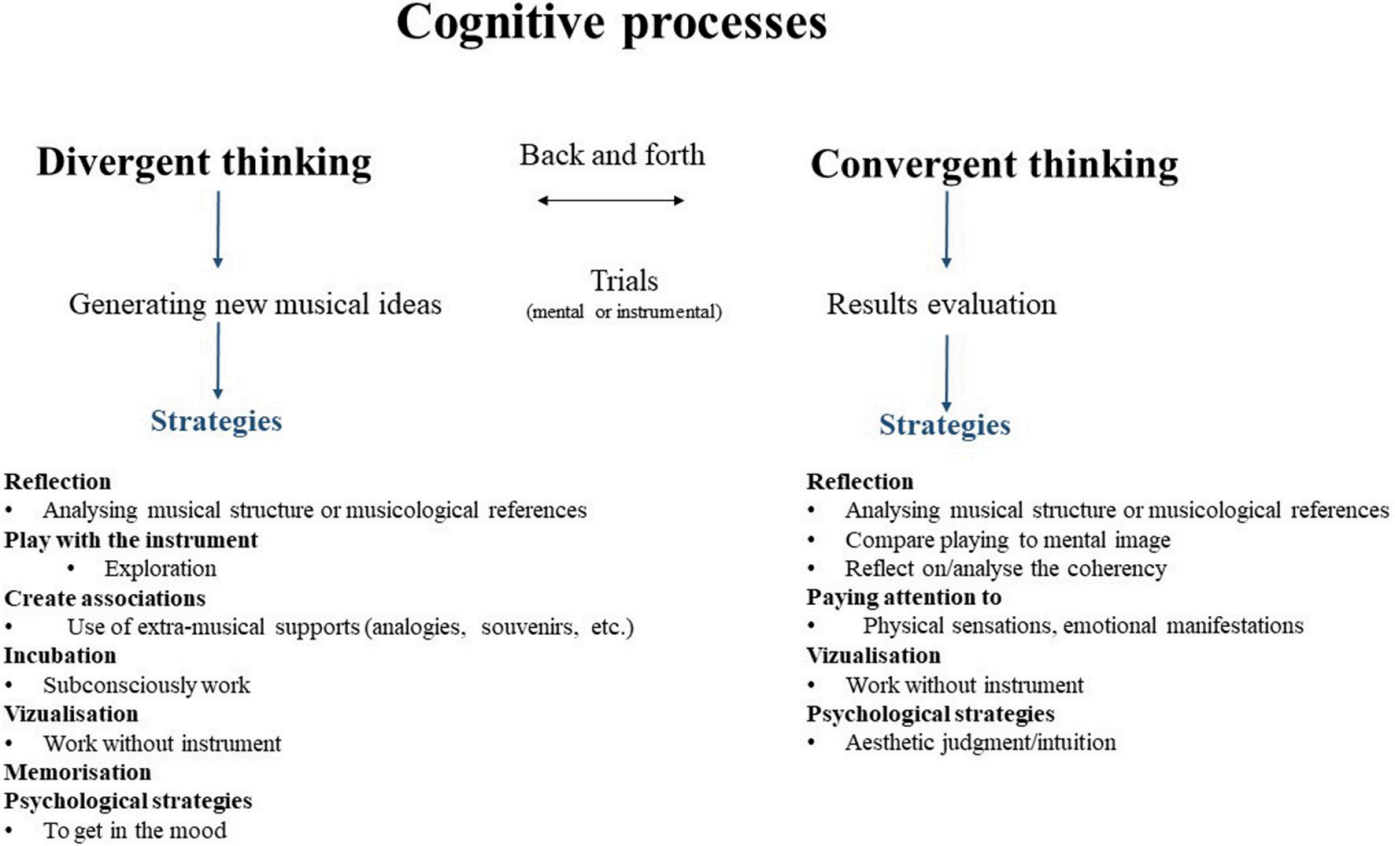
Cognitive processes and strategies observed.

As noted above, five participants heard the music in their head between practice sessions, but only S2, S6, and S7 used these auditory reminiscences (Héroux and Fortier, 2014) as a working strategy to help them refine their artistic image. S6 said, “Since the last practice session, I've been working a little bit on the sound in my head, trying to imagine how I wanted the piece to sound eventually”^27^ (S6.QR.Q1.R3). S9 did not use this strategy consciously, but was convinced that the brain continued to work on its own: “It looks like the brain is still working on it while I'm not working, in the background. Then, when I come back, I have an idea [that is] a little clearer”^28^(S9.EA.7:00). Wallas terms this process “incubation,” whereby “we do not voluntarily or consciously think on a particular problem,” but “a series of unconscious and involuntary (or foreconscious and forevoluntary) mental events may take place” (Wallas, 1926, p. 86). Although we observed this mental process in our study, only two musicians used it consciously as a working strategy to develop their artistic image of the piece. Figure 4 summarizes the cognitive processes and the strategies we observed.

## Limitations

The results presented in this paper provide insight into the creative processes *in situ* but cannot be generalized, given that the research involved only nine participants and the methodology used would not be suitable for a study involving a significantly larger number of participants. A variety of methodologies was used to collect and analyze different types of data. An empirical approach was used to analyze the content of the individual practice sessions of *Why* and to identify work steps, artistic appropriation, and creative moments. We used a phenomenological approach to obtain the participants' points of view regarding their experiences during the individual practice sessions' creative moments and to gather more information on the artistic appropriation stage, both while practicing *Why* and with regard to other musical works. We hoped to identify elements in their backgrounds, practice habits, and values that could explain the choices they made during practice sessions and shed light on parts of their creative processes. Given that empirical and phenomenological research methodologies are separate on the epistemological spectrum, the use of methodologies related to both of them may seem surprising at first. This approach, however, helped us apprehend the complexity of the creative process in shaping an interpretation. The *Analyse par théorisation ancrée* allows the researcher to use a diverse data set in order to explain the complexities of the phenomenon he or she is studying (Paillé, 1994; Paill and Mucchielli, 2008).

The choice of repertoire certainly influenced the outcomes of this study. We tried to control for different factors—length, difficulty level, historical style considerations, prior knowledge of the piece—by imposing the same music on all participants. The results would have been different had we allowed the participants to choose the repertoire freely or to select any work by, say, Bach.

We sought to study the creative process *in situ*, i.e., in the participants' normal environments, but the verbalizations, self-reflection questionnaire, and presence of a camera reminded them that this was a study with a strict protocol. Even so, some data was lost: participants forgot to answer the self-reflection questionnaire in its entirety (S9) or to answer certain questions (S2, S3, S6), or did not record the practice sessions when doing only visualization (S9). Since we chose to let the participants decide when they were ready for recording, we did not control for experiment duration. S7 took 38 weeks and five individual practice sessions (for a total of 87 min and 6 seconds) to go from sight-reading the piece to a final recording, whereas S2 took only 2 weeks (for a total of 105 min and 20 s). These variations probably had an impact on the duration of the work steps, as discussed below.

## Conclusion

To describe the creative process by which expert musicians shape an interpretation is not an easy task. We are grateful to the nine professional musicians who allowed us into the artistic intimacy of their practice sessions with an unfamiliar musical work—*Why*—and accepted the interviewer's intrusive questions. They gave us a unique opportunity to witness performers' creative processes. This in-depth approach allowed us to identify aspects of the creative process that were specific to each individual, as well as elements that all musicians shared.

We found that the context in which the creative process takes place—the musician (e.g., his or her values and knowledge); the musical work (e.g., style, technical aspects, etc.); and the external constraints (e.g., deadlines, public expectations, etc.)—impacted the strategies used. The participants used reflection, extramusical supports, emotions, body reactions, intuition, and other tools to generate new musical ideas and evaluate the accuracy of their musical interpretations.

We identified elements related to those already discussed in the literature, including the creative process as an alternation between divergent and convergent thinking (Guilford, 1950), creative associations (Lubart, 2015), and artistic appropriation (Héroux and Fortier, 2014; Héroux, 2016). We proposed visual representations of the creative process that take into consideration our results regarding how musicians work on music and include context, artistic appropriation, and strategies and processes used.

This research contributes to a better understanding of the creative process underlying performers' work and enhances knowledge in the fields of performance practice and music pedagogy. The outcomes suggest that we must give students solid musical and theoretical tools to make judgments regarding their own musical choices and interpretations. We should encourage students to find their own paths between playing techniques, norms, and originality in interpretation, in order to shape their own creative interpretations.

In the context of this study, the final recordings were only a pretext for the musicians to practice *Why* and develop a professional interpretation for public consumption. This paper only refers to those recordings briefly, to illustrate certain elements of the discussion, though we encourage readers to listen to them in order to hear the sonic results of, say, S1's slow, meditative interpretation or S2's narrative approach, or to contrast S6's dynamic style with S7's more analytic approach. We hope to analyze and discuss these final recordings in more detail in a future paper. For example, to what extent are the nine interpretations of *Why* similar or dissimilar in their expression of the musical structure and in relation to patterns of timing and dynamics? How does this research compare to other studies examining multiple interpretations of a single musical work, such as Chopin's Mazurka Op. 24 No. 2 (Spiro et al., 2010)? Can we identify interpretive norms in our sample, for example in the phrasing decisions made by the musicians or in their deviations from typical approaches to phrasing? The scientific literature offers effective methodologies to analyze these and other acoustic parameters such as vibrato and tone changes (Cook, 2007a; Spiro et al., 2010; Rink et al., 2011; Bisesi and Windsor, 2016). In addition, we hope to analyze the audio-video recordings of the individual practice sessions from a gestural perspective. As noted by Héroux and Fortier (2014), the creation of a mental image and its musical realization may be analyzed through the sound-producing gestures observable from the first reading of a piece through to its interpretation in performance.

Finally, given that the research reported in this article is limited to classical guitarists, the same experiment might be conducted with other instrumentalists and singers in order to determine if they use the same strategies as those identified in the present study. Do other musicians experience the creative process in the same way as our participants did? Further research will enrich our knowledge of the creative processes by which expert musicians shape a musical interpretation.

## Ethics statement

This study was carried out in accordance with the recommendations of the Cadre Normative de la Recherche Avec des Êtres Humaines de l'Université du Québec à Montréal (2012) with written informed consent from all subjects. All subjects gave written informed consent in accordance with the Declaration of Helsinki. The protocol was approved by the Comite Institutionnel d'Éthique de la Recherche Avec des Êtres Humains de l'Université du Québecà Montréal. Certificat number: 2013-S-702927.

## Author contributions

IH is the only researcher on this project and to the writing of this article.

### Conflict of interest statement

The author declares that the research was conducted in the absence of any commercial or financial relationships that could be construed as a potential conflict of interest.

## Footnotes

^2^Please refer to Kozbelt et al. (2010).

^3^Psychologist Pierre Vermersch is the instigator of the explicitation interview technique, which aims to help participants verbalize their actions as lived at a specified time. The interviewer helps the participant remember unconscious actions that are in pre-reflective consciousness. Pre-reflective consciousness, in which we pay attention only to information that appears relevant, is the normal consciousness of our lives. Different aspects of our day-to-day experiences rely on pre-reflective consciousness, as they “are not named by the participant at the moment when they are experienced” (Vermersch, 2010, p. 5).

^4^NVivo is a software program used for qualitative data analysis (videos, interviews, articles, etc.).

^5^The letters refer to interview type and the number or time code gives the location within the interview. EDS: semi-structured interview; EE: explicitation interview; EA: recalled interview.

^6^“Parce que nos connaissances évoluent [et] parce que notre manière de voir les choses évolue.”

^7^“Pour moi c'est beaucoup plus agréable de jouer ces éléments-là quand j'ai un, mon *storyline*, quand j'ai une RAISON de les jouer.”

^8^“Ça c'est plus après. C'est… c'est… c'est une étape plus tard. Moi, quand je suis en studio.”

^9^“[…] dans ce silence-là, y a la préparation physique et mentale du prochain évènement, de la prochaine note.”

^10^“L'homme pensait que c'était correct [ce qu'il a dit], puis la femme dit: ‘non non non.’ […] il essaie de redire… Puis là, elle se choque… Elle a crié — *mais déjà je savais qu'il ne fallait pas qu'elle crie, mon glissando était trop agressif pour le message que je savais que je voulais dire*. […]. Lui [l'homme], il dit: ‘Ah ouain… Je ne savais pas.’ Puis là, il comprend. Les harmoniques [inscrites dans la partition], pour moi c'est la conversation qui arrête, puis ils pensent. C'est comme des petites bulles que l'on voit dans les comics.”

^11^“Là, je commence à trouver les outils nécessaires chez moi pour ME faire pleurer en espérant aussi que le public va pleurer […] Je commence à être ému par ces choses-là que j'ai créées.”

^12^“C'est sûr que le mot *Sorrow* indique […] quelle atmosphère qu'il y a (sic), qui se dégage de cette musique-là, une atmosphère un peu plus… introspective… Peut-être un peu mélancolique.”

^13^“[…] la conjonction de 'Why' et 'Sorrow' est intéressante. […] il y a un élément de tristesse, mais aussi de questionnement, c'est particulier de lier les deux concepts. Cela nous amène à penser à la notion d'énoncé […]c'est comme des pensées un peu éparses, en quelque part. […] Pis ça, ça va avec le questionnement. On se pose des questions, mais se poser des questions, ça ne veut pas dire pas nécessairement avoir un cheminement absolument rectiligne.”

^14^“Être à son service, autant que possible.”

^15^“Je fais ce que je veux, puis si j'ai envie de la jouer de même, je vais la jouer de même.”

^16^“[…] c'est comment moi, je perçois ça, qu'est-ce que ça me fait sentir quand je le joue […]”

^17^“Quand on travaille du répertoire de compositeur ou de période bien connus, on essaye de capter le style ou l'authenticité du style de la pièce […] exemple en musique baroque de respecter les procédures, les procédures d'interprétation baroque.”

^18^“On voit LUI, […] mais on voit aussi la musique.”

^19^“Ça dépend du public.”

^20^“Je cherche en déchiffrant ce que ça amène comme informations musicologiques pour interpréter ça!”

^21^“En général, je te dirais que quand je ne sais pas qu'est-ce que je veux faire ou si j'ai l'impression de jouer mécaniquement, je commence à faire des choses arbitrairement, un peu pour repartir la spontanéité, tsé.”

^22^“[…] parce que ça se rapproche de ce que j'entends dans ma tête […] Je me sens plus près en relation, plus proche de l'idée que j'ai de la pièce, et ce que j'ai exécuté.”

^23^“[…] je la joue, puis si quelque chose spontanément ou intuitivement me vient, ben je vais essayer d'aller valider [avec la partition]. Il y a-tu quelque chose qui supporte ça? Si oui, ben encore là, bingo. Si non, ben on continue à chercher.”

^24^“Fait que c'est ça que je recherche, ici: est-ce qu'on va arriver au point où que dans mon scénario, la femme va crier, ou non? Je suis en train de jouer avec ça.”

^25^“Il fallait que je trouve une façon de me sentir comme ça, nostalgique, c'est une pièce très nostalgique. Pour moi. Et là, j'ai essayé de me rappeler, comme ils font, les method actors, où ils revivent des moments tristes. Et ça m'a beaucoup beaucoup aidé.”

^26^“Cette fin de phrase serait portée à ralentir tout le temps, parce que c'est en rapport avec les deux mots qui sont là, c'est-à-dire ‘sorrow’ et ‘why.’ Donc il y a un aspect interrogateur du fait de ralentir, on n'est plus sûr. On émet quelque chose et après ça, on n'est plus sûr. […] C'est là qu'est le ‘sorrow’ (intervient). C'est cohérent.”

^27^“Depuis la dernière répétition, j'ai travaillé un petit peu sur la sonorité, plus dans ma tête, en essayant d'imaginer comment je voulais que la pièce sonne éventuellement.”

^28^“On dirait que le cerveau continue de travailler sur ça pendant que je ne travaille pas dans le fond. Puis quand je reviens, j'ai une idée un petit peu plus claire.”
